# Viability of internal comparisons for epidemiological research in the US astronaut corps

**DOI:** 10.1038/s41526-023-00278-z

**Published:** 2023-05-12

**Authors:** Robert J. Reynolds, Steven M. Day, Lakshmi Kanikkannan

**Affiliations:** 1 Mortality Research & Consulting, Inc., City of Industry, CA USA; 2 The University of Texas Health Science Center at Houston, Houston, TX USA

**Keywords:** Epidemiology, Statistics

## Abstract

This study aims to determine whether astronauts who have not flown in space can provide an unbiased comparison to astronauts who have flown in space when analyzing long-term health outcomes such as incidence of chronic disease and mortality. Various propensity score methods failed to achieve good balance between groups, demonstrating that even with sophisticated rebalancing methods the group of non-flight astronauts cannot be demonstrated to be an unbiased comparison group for examining the effect of the hazards of spaceflight on incidence and mortality from chronic diseases.

## Introduction

An ongoing challenge for health surveillance among US astronauts is the search for suitable comparison groups. Ironically, this is challenging primarily because astronauts are a remarkably healthy occupational cohort. These results have largely been attributed to the Healthy Worker Effect (HWE), a result of selection and/or survival biases, whereby only initially healthy individuals can join the workforce, and only healthy individuals remain in the workforce over time^1^.

Both the selection bias and survival bias aspects of the HWE are present among US astronauts. The selection bias is intentional: applicants to the US Astronaut Corps must pass rigorous medical selection criteria, which are nearly unparalleled in other occupations. The survival bias stems from the fact that astronauts must maintain health and fitness over the course of their careers as a part of flight readiness. Toward this end, they have access to dedicated astronaut fitness facilities, Astronaut Strength, Conditioning, and Rehabilitation (ASCR) trainers, as well as individualized medical monitoring and care from aerospace medicine physicians and nurses, all at Johnson Space Center in Houston, TX.

In addition to the HWE, US astronauts also benefit from being of moderate-to-high socio-economic status (SES). The pay range for astronauts is over the national median income, and nearly 85% have post-graduate education. Research in the general population has repeatedly demonstrated the survival benefits of both high SES and high levels of education^2^.

In spite of these advantages, it is possible that the unique occupational exposures that come with being an astronaut – particularly exposure to the hazards of spaceflight – may be deleterious to human health. Thus, astronauts who traveled in space may not be as healthy as they might have been had they never gone to space. In essence, the competing forces of the HWE and the unique occupational exposures of spaceflight make it challenging to find a comparison cohort to adequately represent astronauts for the purpose of calculating comparative measures of long-term morbidity and mortality.

Published studies of Astronaut mortality and chronic disease incidence have most often compared rates among astronauts to those of the US general population, but astronauts have also been compared to general population rates adjusted for smoking status^3^, a matched cohort of civil servants at Johnson Space Center^4^, to Soviet-era and Russian Federation cosmonauts^5^, as well as to professional athletes^6^. However, it has been suggested by the scientific community that a natural comparison group might be a subgroup of astronauts themselves, i.e., those who either were selected to the US Astronaut Corps and completed their training but retired from the National Aeronautics and Space Administration (NASA) before completing a spaceflight or are still awaiting a first flight. Such individuals would have the same medical selection criteria imposed upon them, access to the same health maintenance resources from NASA while still employed, and the same SES advantages. Meanwhile, a key difference would be the absence of exposure to space travel. Given these similarities and this difference, this is ostensibly a good comparison. However, while this approach could solve many of the selection bias issues, it could introduce a different set of potential confounders because spaceflight exposure is not randomized. C*onfounding by indication* describes the phenomenon whereby the differential assignment of exposure creates classical confounding: the same factors that determine spaceflight exposure status also are causal factors for health outcomes of interest.

In this exploratory research, we compared those astronauts who retired before achieving their first spaceflight (“no-flight” astronauts) and those who completed at least 1 spaceflight (“flight” astronauts). We fit two different propensity score models to predict spaceflight exposure status, and then used two different types of propensity score weightings to attempt to balance the flight and no-flight groups. For additional comparison, we also performed propensity score matching under two different matching algorithms. We use the standardized mean difference (SMD) score for covariates as the metric of balance between groups.

Summary statistics and the SMDs for the study variables are shown in Table 1, stratified by flight status. The variables we used here are some of those that are predictive of chronic disease incidence and mortality, as well as potentially related to career decisions such as retiring from the Astronaut Corps before taking a space flight. Variables are considered to be balanced between groups when the SMD is less than or equal to 0.1. As such, Table 1 demonstrates that the flight and no-flight groups are balanced on only two characteristics: sex ratio and percentage of astronauts with a history of military service. All other SMDs are greater than 0.1. In particular, educational attainment is severely unbalanced, with 78% of the no-flight group having a doctoral degree and the remaining 22% having a master’s degree, versus only 32% having a doctoral degree, 53% a master’s degree and 15% having only a bachelor’s degree among flight astronauts. Also of note is the 10-year difference in mean year of follow-up start between groups.

**Table 1:** Baseline comparison between flight and no-flight astronauts.

Variable	Flight (*n* = 309)	No-flight (*n* = 9)	SMD
Study start year (mean)	1991.8	1982.8	0.518
Study start age (mean)	40.7	39.2	0.212
Males (%)	86.0	89.0	0.082
Race/ethnicity (%)			0.451
White	90.6	77.8	
Black	4.5	11.1	
Hispanic	3.2	11.1	
Other	1.6	0.0	
Highest Education (%)			1.104
Bachelor	15.2	0.0	
Master	52.8	22.2	
Doctoral	32.0	77.8	
Military (%)	70.0	67.0	0.074
Pilot (%)	70.0	56.0	0.283

*SMD* Standardized Mean Difference.

We first fit a conventional logistic regression model predicting no-flight status, the results of which are shown in Table 2. This standard logistic model largely agreed with the differences in baseline covariates in Table 1 in that the most clearly related factor influencing the risk of retiring before completing a spaceflight was education level, with those who retired before taking a space flight being nearly 15 times more likely to have earned a doctoral degree (MD, PhD, or equivalent). The model also suggested that the risk of retirement before space flight was lower for astronauts selected later in NASA’s history, those selected at older ages, White astronauts, and pilots, while risk of retirement before spaceflight was increased for males and those with a history of military service. However, it should be remembered that the purpose of this model is not to draw inferences about what causes pre-flight retirement but rather to estimate a propensity score for each astronaut, making the interpretation of this model of only secondary interest.

**Table 2:** Comparison of propensity score models predicting flight status.

	Standard Logistic		Firth penalized logistic	
	OR	(95% CI)	OR	(95% CI)
Year of selection	0.93	(0.85, 1.01)	0.94	(0.88, 1.01)
Age at selection	0.92	(0.69, 1.18)	0.94	(0.75, 1.17)
Male	1.62	(0.18, 35.51)	1.33	(0.20, 9.08)
White race	0.16	(0.02, 1.38)	0.19	(0.04, 1.03)
Doctoral degree	14.62	(2.38, 121.37)	12.06	(2.37, 61.41)
Military	6.11	(0.88, 53.72)	4.74	(0.86, 26.15)
Pilot	0.51	(0.06, 3.80)	0.56	(0.09, 3.20)

Using the model to compute propensity scores, we observed that the distribution of predicted propensity scores was almost entirely less than 0.3. Approximately half of the scores were less than 0.01, meaning most astronauts are estimated to have a 1% chance or less of being a no-flight astronaut. These low probability estimates may demonstrate a violation of positivity, a primary assumption of causal inference analysis. Positivity is the assumption that everyone in the sample has a non-zero probability of being exposed; in this case, exposed to spaceflight. In a typical epidemiological study this result may cause a researcher to halt the study and seek a new comparison group, as, philosophically, a violation of the assumption of positivity means that the non-flight and flight groups are not counterfactuals of each other and thus are not comparable. However, here low propensity scores may also be an artifact of a small sample. Since our goal in this research is to compare flight and no-flight astronauts, we were willing to ignore the potential violation of positivity for the sake of fulfilling the study.

Alternative likelihood formulations exist for logistic regression analysis that adjust the model for small numbers of events, such as the small group of no-flight astronauts. One such method is Firth penalized logistic regression, which adds a term that increases the effectiveness of the score function and can adjust in the case of both small and large samples.

**Fig. 1:**
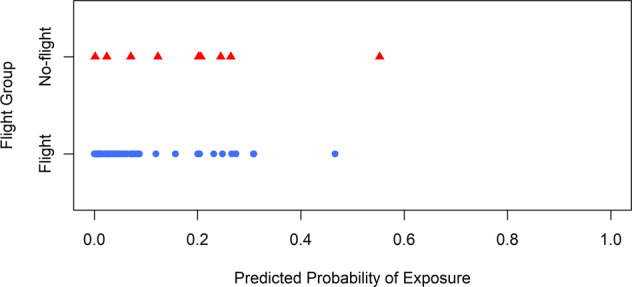
Estimated propensity scores for flight and no-flight astronauts, using a Firth penalized logistic regression model. The estimated scores show that most astronauts have a low probability of going to space, which may suggest a violation of the positivity assumption.

The results of the Firth penalized logistic regression model are also displayed in Table 2. The model is essentially the same as the standard model, though the ORs are all pulled toward 1.0 in comparison to the standard model. Figure 1 shows that, though most propensity scores estimated from this model have been moved toward the middle (i.e., toward 0.5), the bulk of the scores are still below 0.3 with many values again near to zero. As before, for the sake of this demonstration, we proceeded with propensity weighting and balance assessment between the weighted groups ignoring this violation.

Table 3 shows the effective samples sizes and SMDs for the covariates between the flight and no-flight groups under the various weighting schemes as well as for the matched cohorts. Under inverse probability of exposure weighting (IPEW) the effective sample size (ESS) for the standard logistic regression propensity model was inflated to just under 1000 individuals from the original sample size of 318. Similarly, the ESS for the Firth penalized logistic regression using IPEW was just over 670. In comparison to the SMDs in Table 1, we can see that, irrespective of the logistic model used to estimate the propensity scores, the IPEW method worsened the balance in the distribution of most covariates, as evidenced by larger SMD scores than the unweighted sample. Thus, it would seem that IPEW is overall a poor approach, as it threw baseline covariates further out of balance and gave extreme influence to the small numbers of no-flight astronauts.

**Table 3:** Comparison of standardized mean differences of various propensity weighting and matching methods.

	Propensity Weighted				Propensity Matched	
	IPEW		PSOW		Greedy Matching (ESS = 45)	Optimal Matching (ESS = 45)
	Standard Logistic Regression (ESS = 995.67)	Firth penalized Logistic Regression (ESS = 671.62)	Standard Logistic Regression (ESS = 15.20)	Firth penalized Logistic Regression (ESS = 17.87)		
Study start year	1.169	0.955	**0.001**	**0.093**	0.400	0.376
Study start age	1.131	0.940	**0.012**	**0.034**	0.125	0.221
Sex	0.510	0.495	**<0.001**	0.114	**0.081**	**<0.001**
Race/ethnicity	0.431	0.454	0.354	0.356	0.344	0.352
Highest education	1.034	0.919	0.491	0.474	0.317	0.141
Military	0.667	0.572	**<0.001**	**0.051**	0.221	0.221
Pilot	0.762	0.678	**<0.001**	**0.045**	0.161	0.054

Bolded values are considered to be variables in balance (SMD < 0.10).

*IPEW* Inverse probability of exposure weighting, *PSOW* Propensity score with overlap weighting, *SMD* Standardized Mean Difference.

The models using propensity score overlap weighting (PSOW) produced better balance with reasonable ESS. Here the ESS was approximately 15 when using the standard logistic model and approximately 18 when using the Firth penalized logistic regression model. The PSOW method using the standard model brought 5 of the 7 variables into balance, and reduced differences in the remaining two variables. The PSOW method using the Firth penalized model performed nearly as well, bringing 4 of 7 variables into balance.

Overall, propensity matching improved balance between the flight and no-flight groups under both the greedy matching algorithm and the optimal matching algorithm. While matching brought better balance than IPEW, it was not as effective at balancing as PSOW. The greedy and optimal algorithms performed about equally by balancing the same variable and having comparable SMDs for the remaining.

Beyond the obvious issue of group sizes, examination of demographic covariates suggests that there are sizable and potentially important differences between the flight and no-flight groups, which would subject any outcome models to an unknown degree of bias from measured and unmeasured variables. While simple covariate adjustment in outcome models can remove confounding in measured factors, it does nothing to address confounding resulting from unmeasured factors which may be present in non-randomized exposure groups. For example, in the context of the occupation of astronauts, there may be many considerations that lead flight-eligible astronauts to retire from the Astronaut Corps before ever having the opportunity to complete a spaceflight. Some examples of these may be minor health issues that disqualify them from flight eligibility, divergent career ambitions, or changing family situations. These factors, unmeasured here, drive the eventual flight status of astronauts and are reflected by the imbalance in baseline covariates seen in Table 1. Several of these may also exert causal influence on long-term health outcomes.

With the luxury of a larger dataset, the potential for successful propensity analysis would likely improve. Additional astronauts in the sample – particularly no-flight astronauts – would likely lead to more reasonable weights in a propensity-weighted analysis or better matches in the matched analysis. Either of these could, in turn, lead to better balance and hopefully better control of confounding. Thus, the conclusion we draw from this research is that the group of no-flight astronauts is not a valid comparison group for examining the effect of the hazards of spaceflight on long-term health outcomes such as the incidence and mortality of chronic diseases.

This research does not address the potential for flight/no-flight comparisons for outcomes that may be transient and/or observed on much shorter timescales, such as physiological or psychological changes due to spaceflight. However, before attempting to make flight/no flight comparisons in studies of such other outcomes, epidemiologists should still carefully weigh the potential for confounding by indication. The critical question in such instances remains the same: whether or not the outcome is potentially confounded by the non-random nature of spaceflight exposure. As is often the case in research concerning spaceflight, researchers should consider this on a case-by-case basis, within the context of the research question to be answered.

Recent literature has demonstrated that the small size of the Astronaut Corps yields low statistical power in analyses of both incidence and mortality^7,8^. Attempting to analyze even smaller matched sub-groups can only exacerbate this problem. In the 2004 Review of the Longitudinal Study of Astronaut Health, the Committee on the Longitudinal Study of Astronaut Health at the Institute of Medicine wrote, “hypothesis-specific comparison groups will be needed for definitive assessment of specific risks identified in the astronaut”^9^. Nearly 20 years later, we believe that this is still the case.

As humanity continues to plan for the exploration of Mars in the coming decades, understanding the long-term health risks from extended space travel will be more important than ever. Some useful comparisons have already been identified, more are under development, and almost surely even more will be needed. We are confident that with creativity and perseverance, epidemiologists will meet these challenges and help ensure humanity’s destiny among the stars.

## Methods

### Study population

After removing two astronaut candidates (ASCANs) who did not complete the astronaut training, the astronaut dataset comprised 348 astronauts, 39 of whom had not completed a space flight. Subsequent consideration of the data identified three situations that place astronauts in the non-flight group. The most common situation – accounting for 20 of the 39 no-flight astronauts – is that of comparatively recent graduates from astronaut candidacy who are on active duty and awaiting their first flight assignment. Second, there were 10 astronauts who died while on active duty on or before their first spaceflights (9 from vehicular and spacecraft accidents, and one from cancer). The remaining nine no-flight astronauts were those who completed their astronaut training but retired from NASA before receiving a flight assignment.

**Fig. 2:**
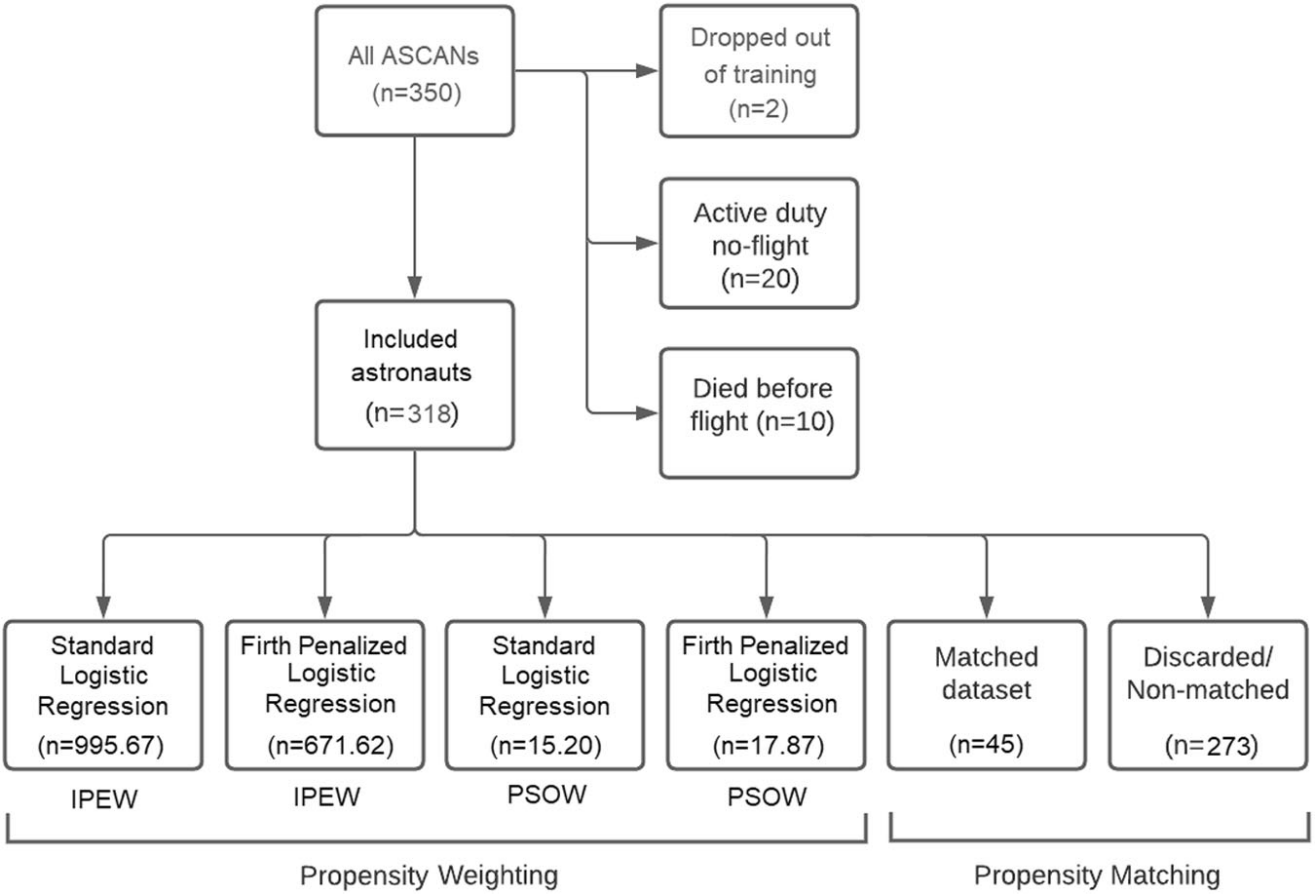
Data cascade diagram for the study of flight and no-flight astronauts. Of the 350 astronauts originally included for study, 32 were disqualified for inclusion based on dropping out of the training class, dying before they could take their first flight, or having as-yet indeterminate flight status. This left 318 astronauts for potential study of long-term health outcomes. Effective sample sizes ranged from a high of 995.67 astronauts (standard logistic regression propensity model with inverse probability of exposure weighting), to a low of 45 astronauts when performing propensity matching.

Death must be considered a competing risk for pre-flight retirement among astronauts waiting to complete their first spaceflights. This means that for flight astronauts, the time from selection as an astronaut candidate to the first spaceflight is *immortal survival time*, or a period in which an astronaut is guaranteed not to have died because he lived long enough to receive the exposure of interest and to be counted among the flight astronauts^10^. Similarly, for non-flight astronauts, the time between selection and retirement from NASA is immortal survival time. This has several important implications for studying health outcomes as a result of spaceflight exposure: (1) the proper follow-up period for assessing the effect of spaceflight on mortality outcomes for flight astronauts begins with their first spaceflight; (2) the proper follow-up period for no-flight astronauts who retired from NASA before completing a spaceflight begins with the date of retirement; (3) astronauts who either are still waiting for their first flight assignment or who died while waiting for their first flight assignment should be considered to have an undefined spaceflight exposure status and should be excluded from analysis. After exclusion of these 30 no-flight astronauts, there were 318 total astronauts left for analysis, 9 of whom were non-flight astronauts. Figure 2 summarizes the data inclusion-exclusion process and the resulting dataset sizes.

### Standardized mean differences

We assessed the balance in covariates between the exposure groups using standardized mean differences (SMD). The SMD for a particular covariate is defined as the difference in means between exposure groups, divided by the pooled standard deviation. When values of the SMD for a particular variable were greater than 0.1 we considered that variable to be imbalanced between groups and interpreted this as evidence of as possible confounding by indication^11^.

### Propensity score models

We next fit two propensity score models and used them to generate two different propensity scores for each astronaut. In this context, the propensity score represents an estimated probability of spaceflight exposure derived from a logistic regression model. The two models were a standard logistic regression model and a Firth penalized logistic regression model. The latter model uses a different likelihood - it adds a term that offsets the first-order term from the expansion of the maximum likelihood estimation. This increases the effectiveness of the score function, and the newly added term goes to zero as the sample size increases, thereby adjusting in the case of both small and large samples^12^.

The models included terms for calendar year of selection, age at selection, sex, race, highest educational attainment (bachelor’s degree or equivalent, master’s degree, or doctoral degree), history of military service, and whether or not the astronaut was a licensed pilot. Because the no-flight group was much smaller than the flight group, we modeled the probability of being in the no-flight group in the propensity score models.

### Inverse probability of exposure weighting (IPEW)

Our first approach to rebalancing the data was to use the propensity score model to generate inverse probability of exposure weights for each observation^13^. In this method, exposed astronauts are assigned a weight of 1/p, and unexposed astronauts are assigned a weight of 1/(1-p), where p is the predicted probability of being in the no-flight group obtained from the propensity score model. In this way, the astronauts who were very unlikely to be in their actual exposure group are up-weighted, and those likely to be in their realized exposure group are down-weighted. This forms a new “pseudo-population” of size equal to the sum of the weights across all astronauts. This weighting allows us to re-examine balance in the pseudo-population and, if appropriate, to use the pseudo-population in outcome analyses.

### Propensity score with overlap weighting (PSOW)

The second form of propensity weighting we used was PSOW^14^. This weighting is computed as (1-propensity score) for members of the treatment group, and simply the propensity score for members of the comparison group. IPEW is widely used but extreme propensities in the study population can lead to bias and unwarranted variance. PSOW is a potential remedy to this problem, as it down-weights the subjects with extreme propensity scores.

### Propensity score matching

Another approach to rebalancing the exposure groups was to conduct propensity score matching. In this method, members of the exposure group are matched to each other according to their propensity score. We used two different matching algorithms here in an attempt to obtain the best possible balance from the matching. First, a nearest-neighbor “greedy” matching was used, followed by “optimal” matching^15^; both are commonly used matching algorithms. Whereas greedy matching takes the best available match for any given observation as it matches each observation in turn, optimal matching attempts to make the best possible set of matches as a whole. These two algorithms often create divergent matched sets, making it worthwhile to implement both here for comparison. The propensity score model used for this matching was the same as the standard logistic regression used for the propensity score weighting described in the previous section.

A review of the literature on propensity matching suggested a ratio of 1 or 2 control subjects to 1 exposed subject is most efficient for minimizing bias, though a ratio of up to 4:1 is considered reasonable^16,17^. As a 2:1 ratio would produce a total sample size of only 27 astronauts, we selected a ratio of 4:1 here. The effect of this choice would be to improve precision at some potential cost of increased bias. Further, as we have only nine available no-flight astronauts in comparison to 309 flight astronauts, we again chose to consider the no-flight astronauts as the “exposed”. This had the effect of guaranteeing that all nine no-flight astronauts were retained in the matched set, as well as exactly 36 for the best-matched flight astronauts, for a total sample size of 45.

### Reporting summary

Further information on research design is available in the Nature Research Reporting Summary linked to this article.

## Supplementary information

Reporting Summary

## Data Availability

The data for this study were taken from our database of astronaut biographical and career details originally compiled from publicly available sources on the internet, such as astronaut biographies on the NASA website, the NASA Astronaut Fact Book, and the popular press articles and obituaries^18,19^. This database has been used extensively in studies of astronaut morbidity and mortality^6,10,20^. This database included information on all US astronauts selected between 1959 and 2017 (NASA astronaut classes 1–22). As the data were gathered from publicly available sources on the internet and contained no protected health information, the study was exempt from institutional review. There are no limitations in accessing the database: for access or for more information regarding the database, please contact Robert Reynolds (rreynolds@mortalityresearch.com).

## References

[1] Fox AJ, Collier PF (1976). Low mortality rates in industrial cohort studies due to selection for work and survival in the industry. J. Epidemiol. Community Health.

[2] Hummer RA, Hernandez EM (2013). The effect of educational attainment on adult mortality in the United States. Popul Bull..

[3] Cucinotta, F. A., Kim, M. H., Chappell, L. J. Space radiation cancer risk projections and uncertainties – 2012. https://three.jsc.nasa.gov/articles/TP_2013_CancerRisk.pdf. Date posted: 02-28-2013.

[4] Hamm PB, Billica RD, Johnson GS, Wear ML, Pool SL (1998). Risk of cancer mortality among the Longitudinal Study of Astronaut Health (LSAH) participants. Aviat., Space, Environ. Med..

[5] Reynolds RJ, Day SM, Nurgalieva ZZ (2014). Mortality of Soviet and Russian cosmonauts, 1960–2013. Aviat. Space Environ. Med..

[6] Reynolds RJ, Day SM (2019). Mortality of US astronauts: comparisons with professional athletes. Occup. Environ. Med..

[7] Reynolds, R. J. Development of a location exposure matrix for ionizing radiation in extraterrestrial environments and its application in the study of mortality for US astronauts. Diss. The University of Texas School of Public Health (2013).

[8] Ade, C. J. et al. Incidence rate of cardiovascular disease end points in the National Aeronautics and Space Administration Astronaut Corps. *J. Am. Heart Assoc*. **6**, (2017). 10.1161/JAHA.117.005564 PMC5586420 28784652

[9] Institute of Medicine. *Review of NASA’s Longitudinal Study of Astronaut Health*. Washington, DC: The National Academies Press. 10.17226/10903 (2004). 25009858

[10] Jones M, Fowler R (2016). Immortal time bias in observational studies of time-to-event outcomes. J. Crit. Care.

[11] Austin, P. C. An Introduction to propensity score methods for reducing the effects of confounding in observational studies. *Multivariate Behav Res*. **46**, 399–424. 10.1080/00273171.2011.568786. 8 (2011). 10.1080/00273171.2011.568786 PMC3144483 21818162

[12] Firth, D. Bias reduction of maximum likelihood estimates. *Biometrika* **80**, 27–38, 10.1093/biomet/80.1.27 (1993)

[13] Cheng, C., Li, F., Thomas, L. Addressing extreme propensity scores in estimating counterfactual survival functions via the overlap weights. (2021). 10.1093/aje/kwac043 35238335

[14] Li F, Morgan KL, Zaslavsky AM (2018). Balancing covariates via propensity score weighting. J. Am. Stat. Assoc..

[15] Austin PC, Stuart EA (2015). Moving towards best practice when using inverse probability of treatment weighting (IPTW) using the propensity score to estimate causal treatment effects in observational studies. Stat. Med..

[16] Austin, P. C. Statistical criteria for selecting the optimal number of untreated subjects matched to each treated subject when using many-to-one matching on the propensity score. *Am. J. Epidemiol.* **172**, 1092–1097. 10.1093/aje/kwq224. Epub 2010 Aug 28 (2010). 10.1093/aje/kwq224 PMC2962254 20802241

[17] Linden A, Samuels SJ (2013). Using balance statistics to determine the optimal number of controls in matching studies. J. Eval. Clin. Pract..

[18] National Aeronautics and Space Administration. NASA Astronaut Factbook. https://www.nasa.gov/pdf/740566main_current.pdf (2005).

[19] Reynolds, R. J., Day, S. M. Mortality among US astronauts: 1980–2009. Aviation, space, and environmental medicine 81.11: 1024–1027 (2010). 10.3357/asem.2847.2010 21043299

[20] Reynolds, R. J. & Day, S. M. The mortality of space explorers. In *Into Space* (ed. Russomano, T.) 253–285 (IntechOpen, London, 2018).

